# Dose- and time- effect responses of DNA methylation and histone H3K9 acetylation changes induced by traffic-related air pollution

**DOI:** 10.1038/srep43737

**Published:** 2017-03-03

**Authors:** Rui Ding, Yongtang Jin, Xinneng Liu, Huaizhuang Ye, Ziyi Zhu, Yuan Zhang, Ting Wang, Yinchun Xu

**Affiliations:** 1 Environmental Epigenetics Laboratory, Department of Environmental Medicine, School of Medicine, Zhejiang University, Hangzhou, Zhejiang, China; 2 Department of Occupational and Environmental Health, School of Public Health, Anhui Medical University, Hefei, Anhui, China; 3 Department of Cardiothoracic Surgery, Sir Run Run Shaw Hospital, School of Medicine, Zhejiang University, Hangzhou, Zhejiang, China; 4 Institute of Pharmacology, Zhejiang University School of Pharmacology, Zhejiang University, Hangzhou, Zhejiang, China

## Abstract

As an important risk factor of respiratory disorders, traffic-related air pollution (TRAP) has caused extensive concerns. Epigenetic change has been considered a link between TRAP and respiratory diseases. However, the exact effects of TRAP on epigenetic changes are still unclear. Here we investigated the dose- and time- effect responses of TRAP on DNA methylations and H3K9 acetylation (H3K9ac) in both blood and lung tissues of rats. The findings showed that every 1 μg/m^3^ increase of TRAP components were associated with changes in %5 mC (95% CI) in *LINE*-*1, iNOS, p16*^*CDKN2A*^, and *APC* ranging from −0.088% (−0.150, −0.026) to 0.102 (0.049, 0.154), as well as 0.276 (0.053, 0.498) to 0.475 (0.103, 0.848) ng/mg increase of H3K9ac. In addition, every 1 more day exposure at high level of TRAP (in tunnel) also significantly changed the levels of DNA methylation (ranging from −0.842% to 0.248%) and H3K9ac (16.033 and 15.718 ng/mg pro in PBMC and lung tissue, respectively) changes. Season and/or sex could interact with air pollutants in affecting DNA methylation and H3K9ac. The findings showed that TRAP exposure is dose- and time- dependently associated with the changes of DNA methylation and H3K9ac.

Air pollution is an important risk factor of respiratory disorders^1^. With the accelerated urbanization and rapid economic development in China, air pollution level also increased rapidly in the past several decades^2^. In addition, car possession increased drastically in China in recent years. Despite the great convenience, the increased number of cars also highlighted a new challenge, namely aggravated traffic-related air pollution (TRAP). Currently, TRAP has been considered as the most important source of air pollution in urban areas^3^. TRAP is a complex mixture of gases (including NO_2_, SO_2_, O_3_) and particulate matters (PMs) with different aerodynamic equivalent diameters^3^,^4^, which could access the respiratory system easily via inhalation, and affect human health directly and indirectly^4^,^5^,^6^. A lot of epidemiological studies have demonstrated that TRAP exposure could increase the risks of respiratory diseases including lung cancer, asthma, and chronic obstructive pulmonary diseases (COPD)^7^,^8^. Time-series studies have further confirmed that the air pollution level is associated with the number of hospitalization and deaths^9^. Even in areas with low levels of air pollution, the exposure could still result in short- and long-term effects on respiratory functions. A recent meta-analysis showed that every 10 μg/m^3^ increase of PM_2.5_ level could increase the risk of lung cancer by 0.09 fold^10^. The time lines of industrialization, worsening of air pollution, and increasing of lung cancer incidence further confirmed the relationship between air pollution and lung cancer. Although TRAP and the health effects are now attracting more and more attentions in China^11^, the specific link between TRAP exposure and respiratory disorders remains incompletely understood.

Epigenetic effects have been universally acknowledged as an important link between environmental factors and health effects^12^. Currently, global DNA methylation, methylation of specific genes, and histone acetylation are the most commonly investigated epigenetic changes^13^. All these changes are heritable and reversible, which occur in early stage of diseases. Previous studies have already demonstrated global hypomethylation and hypermethylation of tumor suppressor genes in lung cancer patients, which could be rescued by effective treatments^14^,^15^. Other studies have also revealed abnormal DNA methylation status in patients with asthma^16^,^17^,^18^ and COPD^19^,^20^. In addition, abnormal histone acetylation (for instance, H3K9ac, H3K18ac, and H4K16ac), which could be rescued by effective treatments, is also associated with respiratory diseases^21^,^22^,^23^. On the other hand, accumulating evidence has shown that air pollutants could potentially induce epigenetic changes. *In vitro* and animal studies have shown that PM exposure is associated with abnormal methylation of long interspersed nucleotide elements-1 (LINE-1) and Alu element^24^. Such changes could be associated with reduced methyl group supply caused by elevated plasma homocysteine level after PM exposure^4^. In addition to global hypomethylation, studies have also shown that air pollutant exposure could induce abnormal methylation of the promoters of specific genes including inducible nitric oxide synthase gene (*iNOS*)^25^,^26^. In addition, PM_10_ level is associated with not only the methylation of *LINE*-*1* and *Alu*, but also the methylation of the promoter of inducible nitric oxide synthase gene, which is closely associated with the development of respiratory diseases^25^. The increase of reactive oxygen species (ROS) level after the TRAP exposure could also increase the iNOS expression, and thus increase the risk of respiratory diseases^25^. A study compared the DNA methylation levels in children from two different regions with different levels of benzene, NO_2_, and PM, and found 9916 CpG sites were significantly different, suggesting that the pollutants including NO_2_ and PM could significantly affect the DNA methylation^26^. In addition, studies have demonstrated that PM exposure could induce the changes of histone acetylation; for instance, NO_2_ and PM could affect the DNA methylation and histone acetylation^27^,^28^,^29^. Therefore, all these findings suggest from both disease and air pollution aspects that epigenetic changes may be an important link between air pollution and respiratory diseases.

Comparing with ambient air pollution, TRAP has several differences in the components of air pollution, percentage of each components, and pollution level. Therefore, it is inappropriate to directly extrapolate the findings in ambient air pollution to TRAP. Several previous studies have already investigated the epigenetic effects of TRAP exposure^5^,^30^,^31^,^32^,^33^,^34^,^35^,^36^,^37^,^38^,^39^,^40^,^41^,^42^; however, there are several drawbacks in those studies. For instance, those studies mainly investigated the effects of single air pollutant, while the effects of TRAP as a mixture were not investigated^5^,^34^,^35^,^36^,^40^; in addition, most studies investigated epigenetic effects in different white blood cells^5^,^30^,^31^,^32^,^33^,^34^,^37^,^38^,^40^,^41^,^42^, which represent the overall results from all the organs of the whole body, while the effects in the target organ, namely lung, were not been well studied; furthermore, ambient air monitoring data were from central air monitoring stations adopted in most studies to estimate the air pollution level^30^,^32^,^33^,^36^,^39^,^41^,^42^, which may not loyally represent the TRAP level.

In the present study, Wistar rats were exposed at different traffic sites for 4 h, 7 d, 14 d, and 28 d, respectively. Then global DNA methylation, methylation of 3 gene-specific genes, and acetylation of histone H3K9 were measured in both blood and lung tissues to investigate the epigenetic effects of TRAP exposure.

## Results

### DNA methylation and histone H3K9ac after 4 h and 7 d exposure

No significant difference in the DNA methylation and H3K9ac levels were found between spring and autumn in this study, thus the data in the two seasons were combined for analysis. *LINE*-*1* and *iNOS* promoter methylation levels were significantly lower, while *p16*^*CDKN2A*^ and *APC* promoter methylation levels were significantly higher in tunnel and/or crossroad groups than in control group in 4 h and 7 d exposure windows; H3K9ac level was not significantly different in 4 h window, but was significantly higher in tunnel and crossroad groups than in control groups in 7 d window (See Supplementary Fig. 1).

### Dose-effect responses

Levels of air pollutants at the 3 exposure sites are shown in Supplementary Fig. 2 (See Supplementary Fig. 2). As significant correlations were found among PM_2.5_, PM_10_, an NO_2_ levels in this study (all *P* values < 0.001), they were included separately into the regression model along with temperature, season, and SO_2_ level as the independent variables, to investigate the dose-effect responses with DNA methylation and H3K9ac levels.

In 4 h exposure window, every 1 μg/m^3^ increase of PM_2.5_ resulted in 0.03% and 0.04% decrease of *LINE*-*1* and *iNOS* methylation in DNA from blood, and 0.04% decrease of *LINE*-*1* methylation in DNA from lung tissues, respectively. Every 1 μg/m^3^ increase of PM_10_ resulted in 0.02% and 0.02% decrease of *LINE*-*1* and *iNOS* methylation in DNA from blood, and 0.03% decrease of *LINE*-*1* methylation in DNA from lung tissues, respectively. Every 1 μg/m^3^ increase of NO_2_ resulted in 0.08% and 0.10% increase of *iNOS* methylation in DNA from blood and lung tissues, and 0.09% decrease of *p16*^*CDKN2A*^ promoter methylation in DNA from blood, respectively (Table 1).

In 7 d window, every 1 μg/m^3^ increase of PM_2.5_ resulted in 0.06% and 0.05% decrease of *LINE*-*1* and *iNOS* methylation in DNA from blood, 0.03% and 0.05% decrease of *LINE*-*1* and *iNOS* methylation in DNA from lung tissues, 0.01% increase of *APC* promoter methylation in DNA from lung tissues, and 0.48% and 0.47% increase of H3K9ac from PBMC and lung tissues, respectively. Every 1 μg/m^3^ increase of PM_10_ resulted in 0.04% and 0.02% decrease of *LINE*-*1* methylation in DNA from blood and lung tissues, 0.03% decrease of *iNOS* methylation in DNA from lung tissues, and 0.28% and 0.28% increase of H3K9ac from PBMC and lung tissues, respectively. Every 1 μg/m^3^ increase of NO_2_ resulted in 0.09% increase of *LINE*-*1* methylation in DNA from blood, and 0.10% increase of *iNOS* methylation in lung tissues, respectively (Table 1).

The dose-effect responses in the rats exposed in tunnel for different time also confirmed the above findings. After sex, season, SO_2_ level, temperature, and age were adjusted, the results showed that every 1 μg/m^3^ increase of PM_2.5_ resulted in 0.04% increase of *p16*^CDKN2A^ promoter methylation in blood DNA, 0.02% and 0.06% decrease of *LINE*-*1* and *iNOS* methylation in lung tissue DNA, 0.01% and 0.01% increase of *APC* and *p16*^CDKN2A^ promoter methylation in lung tissue DNA, and 0.64 and 0.66 ng/mg protein increase of H3K9ac from PBMC and lung tissue histone, respectively. Every 1 μg/m^3^ increase of PM_10_ resulted in 0.01% and 0.02% decrease of *LINE*-*1* and *iNOS* methylation, and 0.029% increase of *p16*^CDKN2A^ promoter methylation in blood DNA, 0.02% decrease of *iNOS* promoter methylation in lung tissue DNA, and 0.18 and 0.19 ng/mg protein increase of H3K9ac from PBMC and lung tissue histone, respectively. Every 1 μg/m^3^ increase of NO_2_ did not result in significant changes of blood DNA methylation, but resulted in 0.07% decrease of *LINE*-*1* methylation and 0.07% increase of *iNOS* methylation in lung tissue DNA.

### Time-effect responses

**Figure 1:**
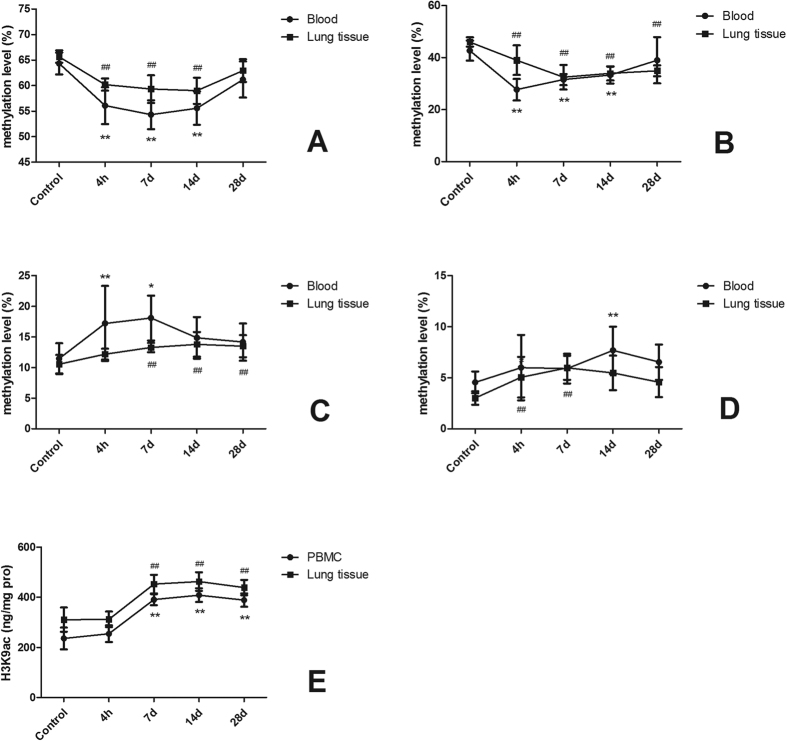
Comparison of DNA methylation and H3K9ac after exposed for 4 h, 7 d, 14 d, and 28 d in tunnel. The data of the control groups were the mean values in the rats exposed in the control site for 4 h. A, *LINE*-*1*; B, *iNOS*; C, *p16*^*CDKN2A*^; D, *APC*; and E, H3K9ac. *P < 0.05, **P < 0.01, comparing with control group in blood samples. ^#^*P* < 0.05, ^##^*P* < 0.01, comparing with control group in lung tissue samples.

We firstly compared the DNA methylation and H3K9ac levels of the rats exposed for 4 h, 7 d, 14 d, and 28 d in the tunnel. The results showed that the methylation of *LINE*-*1, iNOS, p16*^CDKN2A^, and *APC* changed evidently with time, but recovered more or less after exposed for 28 d (Fig. 1). Therefore, we excluded the data from 28 d exposure, and then evaluated the time-effect responses. The results showed that after age, sex, season, body weight, lung weight, and temperature were adjusted, exposure in the tunnel for 1 more day resulted in significant decrease of *LINE*-*1* (by 0.61% in blood DNA and 0.32% in lung tissue DNA) and *iNOS* promoter (by 0.84% in blood DNA and 0.69% in lung tissue DNA) methylation, and significant increase of *p16*^*CDKN2A*^ promoter methylation in lung tissue DNA (by 0.25%) (Table 2).

In contrast to DNA methylation, the comparison of H3K9ac levels of rats exposed for different time in the tunnel showed that H3K9ac in both PBMC and lung tissue histones increased with time. The further analysis with the data from all 4 exposure windows showed that after age, sex, season, body weight, lung weight, and temperature were adjusted, exposure in the tunnel for 1 more day resulted in 16.03 and 15.72 ng/mg pro increase of H3K9ac in histone from PBMC and lung tissues, respectively (Table 2).

### Interactions of season and sex with air pollutants

Generalized linear model was used to explore the interactions of season and sex with air pollutants in affecting DNA methylation and H3K9ac. The results showed that there were significant interactions between season (but not sex) and PM_2.5_, NO_2_, and SO_2_ in affecting H3K9ac (Table 3).

Season could interact with PM_2.5_, PM_10_, and NO_2_ in affecting blood LINE-1 methylation, interact with PM_10_ and NO_2_ in affecting blood *p16*^*CDKN2A*^ promoter methylation, interact with PM_2.5_ in affecting lung tissue *LINE*-*1* methylation, and interact with PM_10_ in affecting lung tissue *p16*^*CDKN2A*^ promoter methylation. Sex could interact with PM_2.5_ in affecting blood *APC* promoter methylation, interact with PM_10_ in affecting lung tissue *LINE*-*1* methylation, and interact with PM_2.5_ in affecting lung tissue *p16*^*CDKN2A*^ promoter methylation (Table 3).

### Associations between DNA methylation and H3K9 acetylation

As shown in Table 4, PBMC H3K9ac was negatively associated with blood *LINE*-*1* methylation, and positively associated with blood *APC* promoter methylation. In contrast to the associations in blood, the findings in lung tissues showed that H3K9ac was negatively associated with both *LINE*-*1* and *iNOS* methylation, and positively associated with *APC* and *p16*^*CDKN2A*^ promoter methylation.

## Discussion

In the present study, rats were exposed at the roadside of a crossroad and a tunnel for different time, and the findings revealed that TRAP exposure could dose- and time- dependently affect DNA methylation and H3K9ac in both blood and lung tissues. To our knowledge, this is the first study investigating the effects of TRAP on DNA methylation in both blood and lung tissues.

Accumulating evidence shows altered DNA methylation and histone acetylation could occur in extremely early stages of COPD, asthma, and lung cancer^43^. *LINE*-*1* is an important repetitive sequence that accounts for about 17% of the length of human genome^44^. During the process of DNA methylation, about one third of methylations occur at repetitive sequences^45^. Previous studies have shown that *LINE*-*1* methylation is closely associated with global methylation level^46^; therefore *LINE*-*1* methylation has been widely used to reflect global methylation level. Decreased *LINE*-*1* methylation is associated with genome instability and gene expression dysregulation^43^, and has been considered as one important feature of pulmonary diseases^47^. The findings of the present study showed that even rapid exposure (4 h) to TRAP could effectively reduce *LINE*-*1* methylation in both blood and lung tissue DNA, while the decrease after 7 d exposure was more evident. The dose-effect responses between air pollutants and *LINE*-*1* methylation strongly suggest that global hypomethylation could occur in early exposure stage, and is very sensitive to air pollution changes. As dose-dependent *LINE*-*1* hypomethylation was still found in the 7 d exposure window, as well as prolonged exposure in the tunnel, we speculate that the global hypomethylation induced by TRAP exposure is not just a transient change induced by acute stress, but a relatively stable change that could affect the genome stability and activation of oncogenes, which may finally increase the risk of lung tumors. However, in another study in welders^48^, investigators found that occupational exposure of PM_2.5_ did not result in significant change in *LINE*-*1* methylation. The differences with our findings could be associated with the differences of pollution components. For instance, PM_2.5_ for welders contains high levels of metals^49^, while TRAP contains relatively low levels of metals but higher levels of carbon black and other gases^50^. In addition, the protective equipment of the workers could also provide substantial protective effects.

The findings of the present study showed that TRAP exposure significantly reduced *iNOS* promoter methylation. However, the change in lung tissue DNA was slightly different from the change in blood DNA. For instance, 4 h exposure in both tunnel and crossroad significantly reduced *iNOS* promoter methylation in blood DNA, but the lung tissue DNA showed no significant decrease in *iNOS* promoter methylation. In contrast to the changes in 4 h window, 7 d exposure at both crossroad and tunnel, and prolonged exposure in tunnel significantly reduced *iNOS* promoter methylation. The dose-effect relationship between air pollutant levels and *iNOS* methylation also confirmed these findings. These findings suggest that the methylation of *iNOS*, a gene closely associated with systemic inflammation, is very sensitive to TRAP exposure. However, rapid TRAP exposure may mainly affect systemic inflammation, while inflammation in lung tissues may not be as sensitive to TRAP exposure, which could be associated with the protective effects from nasal cavity and upper respiratory tract. In addition, these findings also suggest that in early exposure phase, *iNOS* methylation in peripheral blood DNA may not accurately reflect the changes in lung tissues. However, prolonged TRAP exposure could affect not only *iNOS* methylation in blood DNA but also lung tissue DNA. Therefore, our findings suggest that TRAP exposure could cause *iNOS* gene hypomethylation. Such alteration could lead to increased *iNOS* expression, which in turn increase NO and ONOO^−^ levels^51^, and therefore affect DNMTs to influence global DNA methylation^52^.

The findings of the present study also showed that TRAP exposure could increase *p16*^*CDKN2A*^ and *APC* gene promoter methylation. *p16*^*CDKN2A*^ is a tumor suppressor gene, which encode P16 protein to play important roles in cell cycle regulation. Previous studies have shown that the rate of *p16*^*CDKN2A*^ gene silencing is about 67% in lung adenocarcinoma, and is as high as 70% in squamous cell carcinomas^53^,^54^,^55^,^56^. However, silencing of *p16*^*CDKN2A*^ gene is mainly caused by promoter hypermethylation instead of gene mutation^53^. The findings of the present study showed that *p16*^*CDKN2A*^ gene promoter methylation did not change significantly after 4 h exposure. However, 7 d exposure at the tunnel but not crossroad significantly increased *p16*^*CDKN2A*^ gene promoter methylation. These findings suggest that rapid TRAP exposure may not be sufficient to increase *p16*^*CDKN2A*^ gene promoter methylation, which could be associated with the powerful clearance ability of the respiratory system. In contrast, prolonged TRAP exposure could effectively induce *p16*^*CDKN2A*^ gene hypermethylation, thus reduce *p16*^*CDKN2A*^ gene expression, affect cell cycle, and finally play an important role in the development of multiple pulmonary diseases. The present study showed no significant change in *APC* promoter methylation in blood DNA after 4 h and 7 d exposure. In contrast, both 4 h and 7 d TRAP exposure in tunnel significantly increased *APC* promoter in lung tissues. These findings indicate that methylation of *APC* from other tissues may mask the change in lung tissues, thus no significant *APC* methylation could be detected in blood DNA.

The rats were further exposed in the tunnel for 14 and 28 d to investigate the time-effect responses of TRAP exposure on DNA methylation and H3K9ac. The results showed that DNA methylation and H3K9ac changed significantly with the increase of exposure time. However, the changes of DNA methylation in lung tissue were not always consistent with the changes in blood DNA. We speculated that the follows should be considered while interpreting the findings: 1) after prolonged exposure to TRAP, DNA methylation in blood and lung tissue DNA may be different; therefore the value of using methylation level in blood DNA to help disease diagnosing and outcome predicting may be limited, which further underscored the importance of measuring DNA methylation in lung tissues; and 2) although long term exposure may not necessarily induce continuous DNA methylation changes, the methylation may still remain at abnormal level and thus increase the risk of multiple diseases.

Although the importance of both DNA methylation and H3K9ac in the development of pulmonary diseases has already been widely acknowledged, these events need to interact with each other to finally increase the risks of pulmonary diseases. On the other hand, interactions between DNA methylation and histone acetylation are critical for maintaining normal development. The findings of the present study showed that histone H3K9ac increased significantly after TRAP exposure, also in dose- and time- dependent manner. In addition, H3K9ac increase was associated with global hypomentylation and hypermethylation of *p16*^*CDKN2A*^ and *APC*, especially in lung tissues. These findings indicate histone acetylation is closely correlated with DNA methylation, which could interact with each other and finally increase the risk of pulmonary diseases including lung cancer.

There are several limitations in this study. First, the study only focused on describing the effects of TRAP on the epigenetic marks in both rat blood and lung tissues, while the consequences of such changes were not investigated. Second, although 96 rats were used in this study, the sample size is still relatively small, especially when the analyses were performed after the influencing factors were adjusted. Third, controls were only used for the 4 h and 7 d exposure windows in this study. Although the prolonged exposures (14 d and 28 d) in the tunnel were mainly used for the investigation of time-effect response, controls for these two exposure windows could help explaining the results. Finally, the exact components of the particulate matters were not measured in this study, and thus the difference in the components at the 3 exposure sites could not be clarified, which could at least partially affect the epigenetic changes.

In summary, the present study firstly exposed the rats at the sites of traffic-related air pollution to investigate the early effects, cumulative effects, dose-effect responses, and interactions of the air pollution in affecting DNA methylation and H3K9 acetylation. The findings showed that the early effect of traffic-related air pollution exposure mainly included global DNA hypomethylation and changes in the inflammation-related gene, while prolonged exposure could effectively affect the methylation of specific genes; high level of traffic-related air pollution exposure had significant cumulative effects on DNA methylation and H3K9 acetylation in a certain range of exposure time, and then tended to recover; season and sex could interact with air pollutants in affecting DNA methylation and H3K9 acetylation.

## Materials and Methods

### Study design

**Figure 2:**
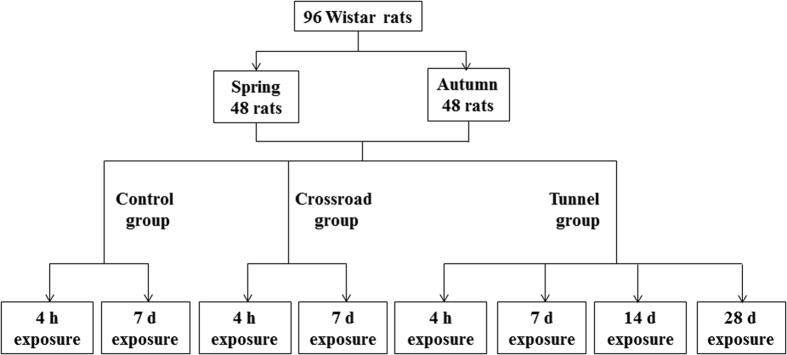
Schematic diagram of the process of exposure in this study.

This study was performed in both spring and autumn. In each season, 48 Wistar rats (24 males and 24 females, Shanghai Slac Laboratory Animal Co., Ltd., Shanghai, China) with the ages of 8 weeks were randomly divided into 8 groups using the random number table. The rats underwent 1 week of acclimatization before the experiments. The study adhered to the US National Institutes of Health guidelines for the use of experimental animals. The animal care and handling procedures were approved by the Institutional Animal Care and Use Committee of Zhejiang University. The exposure processes of this study are shown in Fig. 2.

The rats were exposed at 3 sites with different TRAP levels, namely tunnel, crossroad, and control, for 4 h, 7 d, 14 d, and 28 d, respectively. The exposure sites were selected according to our previous preliminary study. The tunnel selected in this study was the Lingxi Tunnel (Hangzhou, China). This tunnel is 1492 m long and composes of 4 lanes. The traffic flow in this tunnel is about 1500–2200 vehicles/hour generally, and over 3000 vehicles/hour at peak time (7:00–8:00 and 18:00–19:30 on working days). The crossroad selected in this study is at the intersection of two busy roads (namely Wenyixi and Gudun roads) in Hangzhou, China. The traffic flow at the crossroad is about 2000–3000 vehicles/hour generally, and over 4500 vehicles/hour at peak time. Traffic jams are very common in the tunnel and at the crossroad. The west-north campus of Zhejiang University that covered by trees and grass is selected for exposure of the control group, from where the nearest road for cars is more than 0.5 mile away. For the exposure, male and female rats were kept separately in polypropylene cages with three rats per cage. For the rats exposed for 4 h, the exposure was started at 7:30 and ended at 11:30; while for the rats exposed for 7, 14, and 28 d, the exposure was started at 7:30 and ended at 19:30 for 7, 14, and 28 continuous days, respectively.

### TRAP assessment

As reported in our previous studies^47^,^49^, middle volume total suspended particulates sampler (ZC-Q0101, Hengda Instrument Company, Zhejiang, China) was used to estimate the levels of PM_2.5_ and PM_10_ (PM with aerodynamic diameter <10 μm) once every hour during the exposure according to the manufacturer’s instructions. Saltzman method was used to measure the NO_2_ level, and hydrochloric pararosaniline spectrophotometry was used to measure the SO_2_ level. Temperature was also recorded once every hour during the exposure.

### Sample preparations

After the exposure, the rats were brought back to the laboratory immediately, anesthetized by CO_2_ inhalation, and then blood was collected from the femoral artery. The rats were then sacrificed by cervical dislocation. The lungs of the rats were removed, washed with ice-cold saline, blotted dry, and stored at −80 °C until use.

### DNA methylation and H3K9 acetylation quantification

Genomic DNA was extracted from both the blood and lung tissues using DNA extraction kits (TIANamp Genomic DNA kit, Cat. DP304-03; Tiangen, Beijing, China). The global methylation (*LINE*-*1*) and methylation of the promoters of gene-specific genes (*iNOS, p16*^*CDKN2A*^, and *APC*) were quantified by PCR-pyrosequencing. The level of methylation was described as percentage of methylated cytosine (%5 mC) divided by the sum of methylated and unmethylated cytosine measured in each individual sample.

Histones were extracted from lung tissues and peripheral blood mononuclear cells (PBMCs), using the EpiQuik total histone extraction kit (Epigentek group). The concentration of total histone was quantified with the bicinchoninic acid assay kit (Maibio). The histone H3K9 acetylation in the peripheral blood mononuclear cells (PBMCs) and lung tissues was measured with the EpiQuik TM Global acetyl histone H3K9 quantification kit (Epigentek group) using microplate absorbance reader (Infinite F50; TECAN, Grodig, Austria). The level of H3K9 acetylation was described as ng/mg pro.

### Statistical analysis

SPSS19.0 software was used for the statistical analysis, and GraphPad Prism 5 software was used for the chart plotting. The quantitative data were described with means and standard divisions, and compared with one-way analysis of variance (ANOVA). General linear model was used to explore the interactions of season and sex with the pollutant concentration in inducing the DNA methylation and H3K9 acetylation changes. Multiple linear regression was applied to assess the dose- and time- effect responses of the air pollutants on DNA methylation and H3K9 acetylation levels. P ≤ 0.05 was considered statistically significant.

## Additional Information

**How to cite this article:** Ding, R. *et al*. Dose- and time- effect responses of DNA methylation and histone H3K9 acetylation changes induced by traffic-related air pollution. *Sci. Rep.* **7**, 43737; doi: 10.1038/srep43737 (2017).

**Publisher's note:** Springer Nature remains neutral with regard to jurisdictional claims in published maps and institutional affiliations.

## Supplementary Material

Supplementary Information

## Figures and Tables

**Table 1:** Dose-effect responses between air pollutants and DNA methylation and H3K9ac [β (95% CI)].

		4 h		7 d	
		Blood	Lung tissue	Blood	Lung tissue
PM_2.5_	*LINE*-*1*	−0.03 (−0.04, −0.01)**	−0.04 (−0.05, −0.03)**	−0.06 (−0.10, −0.02)**	−0.03 (−0.06, −0.01)*
	*iNOS*	−0.04 (−0.06, −0.02)**	−0.02 (−0.04, 0.01)	−0.05 (−0.09, −0.00)*	−0.05 (−0.09, −0.02)**
	*p16*	0.02 (−0.01, 0.04)	0.01 (−0.002, 0.01)	−0.01 (−0.03, 0.03)	0.01 (−0.00, 0.03)
	*APC*	0.01 (−0.01, 0.03)	−0.00 (−0.01, 0.01)	0.01 (−0.01, 0.02)	0.01 (0.00, 0.02)*
	H3K9ac	0.04 (−0.22, 0.31)	−0.03 (−0.34, 0.28)	0.48 (0.10, 0.85)**	0.47 (0.09, 0.85)**
PM_10_	*LINE*-*1*	−0.02 (−0.03, −0.01)**	−0.03 (−0.03, −0.02)**	−0.04 (−0.06, −0.01)**	−0.02 (−0.03, −0.01)*
	*iNOS*	−0.02 (−0.04, −0.01)**	−0.01 (−0.02, 0.002)	−0.03 (−0.06, 0.01)	−0.03 (−0.05, −0.01)**
	*p16*	0.01 (−0.01, 0.02)	0.00 (−0.01, 0.01)	−0.01 (−0.02, 0.02)	0.01 (−0.00, 0.02)
	*APC*	0.01 (−0.01, 0.02)	0.00 (−0.01, 0.00)	0.01 (−0.01, 0.01)	0.01 (0.00, 0.01)
	H3K9ac	0.03 (−0.15, 0.20)	−0.02 (−0.22, 0.18)	0.28 (0.06, 0.50)**	0.28 (0.05, 0.50)**
NO_2_	*LINE*-*1*	0.02 (−0.02, 0.06)	0.01 (−0.01, 0.04)	0.09 (0.02, 0.17)*	0.05 (−0.00, 0.10)
	*iNOS*	0.08 (0.02, 0.13)*	0.10 (0.05, 0.15)**	0.05 (−0.05, 0.16)	0.10 (0.03, 0.17)**
	*p16*	−0.09 (−0.15, −0.03)**	−0.02 (−0.05, 0.002)	0.02 (−0.03, 0.08)	−0.02 (−0.05, 0.01)
	*APC*	−0.01 (−0.05, 0.04)	−0.00 (−0.02, 0.02)	0.01 (−0.03, 0.03)	−0.01 (−0.03, 0.01)
	H3K9ac	−0.07 (−0.73, 0.63)	0.05 (−0.82, 0.92)	−0.55 (−1.26, 0.15)	−0.49 (−1.21, 0.23)

Abbreviations: CI, confidential interval; PM_2.5_, particulate matter with aerodynamic diameter <2.5 m; PM_10_, particulate matter with aerodynamic diameter <10 m; *LINE*-*1*, long interspersed nucleotide element; *iNOS*, inducible nitric oxide synthase; *APC*, adenomatous polyposis coli; H3K9ac, H3K9 acetylation.

*P < 0.05, **P < 0.01, adjusted for temperature, season, and SO_2_ level.

**Table 2:** Time-effect responses between air pollutants and DNA methylation and H3K9ac [β (95% CI)].

	Blood	Lung tissue
*LINE*-*1*	−0.61 (−1.37, −0.16)*	−0.32 (−0.64, −0.01)*
*iNOS*	−0.84 (−1.22, −0.46)*	−0.69 (−1.19, −0.19)*
*p16*	−0.01 (−0.42, 0.40)	0.25 (0.11, 0.39)**
*APC*	0.22 (−0.12, 0.55)	−0.02 (−0.21, 0.18)
*H3K9ac*	16.03 (12.78, 19.29)**	15.72 (10.55, 20.88)**

Abbreviations: CI, confidential interval; *LINE*-*1*, long interspersed nucleotide element; *iNOS*, inducible nitric oxide synthase; *APC*, adenomatous polyposis coli; H3K9ac, H3K9 acetylation.

*P < 0.05, **P < 0.01. Adjusted for age, sex, season, body weight, lung weight, and temperature.

**Table 3:** Interactions of season and sex with air pollutants in affecting DNA methylation and H3K9ac (β, 95% CI).

	*LINE*-*1*	*iNOS*	*p16*	*APC*	H3K9ac
**Blood**					
Season * PM_2.5_	−0.388 (−0.571, −0.206)**	−0.200 (−0.512, 0.112)	0.085 (−0.118, 0.288)	0.061 (−0.055, 0.178)	1.30 (0.88, 1.71)**
Season * PM_10_	0.121 (0.001, 0.240)*	0.011 (−0.193, 0.215)	−0.192 (−0.325, −0.059)**	−0.015 (−0.091, 0.062)	0.25 (−0.15, 0.65)
Season * NO_2_	−0.739 (−1.398, −0.080)*	−0.435 (−1.559, 0.689)	0.975 (0.242, 1.707)**	−0.137 (−0.556, 0.283)	−7.52 (−9.65, −5.39)**
Season * SO_2_	10.183 (−4.995, 11.370)	9.097 (0.252, 17.942)	−0.324 (−6.088, 5.440)	−0.752 (−4.053, 2.550)	31.58 (23.21, 39.95)**
Sex * PM_2.5_	−0.080 (−0.183, 0.022)	0.147 (−0.027, 0.322)	0.053 (−0.060, 0.167)	0.086 (0.020, 0.151)*	−0.08 (−0.32, 0.16)
Sex * PM_10_	0.021 (−0.074, 0.115)	0.022 (−0.139, 0.184)	−0.079 (−0.184, 0.026)	−0.037 (−0.098, 0.023)	0.09 (−0.04, 0.216)
Sex * NO_2_	0.584 (−0.098, 1.265)	−0.733 (−1.895, 0.428)	−0.121 (−0.878, 0.636)	−0.015 (−0.449, 0.418)	−0.43 (−1.13, 0.26)
Sex * SO_2_	0.838 (−1.516, 3.192)	−3.143 (−7.156, 0.870)	−0.072 (−2.687, 2.543)	−1.681 (−3.179, −0.184)	3.34 (−1.08, 7.76)
**Lung tissue**					
Season * PM_2.5_	−0.145 (−0.287, −0.004)*	−0.137 (−0.326, 0.051)	0.042 (−0.035, 0.119)	−0.057 (−0.115, 0.000)	1.45 (0.93, 1.98)**
Season * PM_10_	0.074 (−0.019, 0.167)	0.039 (−0.084, 0.163)	−0.058 (−0.108, −0.007)*	−0.001 (−0.039, 0.036)	0.17 (−0.38, 0.67)
Season * NO_2_	0.093 (−0.418, 0.604)	−0.109 (−0.788, 0.571)	0.190 (−0.088, 0.468)	0.116 (−0.092, 0.323)	−7.76 (−10.45, −5.07)**
Season * SO_2_	1.502 (−2.517, 5.521)	1.186 (−4.162, 6.534)	−0.675 (−2.862, 1.512)	1.877 (−0.103, 3.511)	32.40 (21.81, 42.98)**
Sex * PM_2.5_	−0.046 (−0.125, 0.033)	−0.114 (−0.220, −0.009)*	0.064 (0.021, 0.107)**	0.003 (−0.030, 0.035)	−0.24 (−0.54, 0.07)
Sex * PM_10_	0.064 (−0.009, 0.137)	−0.031 (−0.128, 0.066)	−0.004 (−0.044, 0.036)	−0.002 (−0.032, 0.028)	0.14 (−0.02, 0.30)
Sex * NO_2_	−0.194 (0.333, 0.521)	0.446 (−0.256, 1.148)	−0.008 (−0.295, 0.279)	0.121 (−0.093, 0.336)	0.20 (−0.67, 1.08)
Sex * SO_2_	−0.337 (−1.486, 0.131)	2.109 (−0.317, 4.535)	−1.314 (−3.306, 0.322)	0.490 (−0.251, 1.231)	−0.90 (−6.49, 4.69)

Abbreviations: CI, confidential interval; PM_2.5_, particulate matter with aerodynamic diameter <2.5 m; PM_10_, particulate matter with aerodynamic diameter <10 m; *LINE*−*1*, long interspersed nucleotide element; *iNOS*, inducible nitric oxide synthase; *APC*, adenomatous polyposis coli; H3K9ac, H3K9 acetylation.

*P < 0.05, **P < 0.01, adjusted for temperature.

**Table 4:** Associations between H3K9ac and DNA methylation induced by TRAP exposure.

	LINE-1		*iNOS*		*APC*		*p16*^*CDKN2A*^	
	*r*^*2*^	*P*	*r*^*2*^	*P*	*r*^*2*^	*P*	*r*^*2*^	*P*
Blood	−0.391	<0.001	−0.199	0.053	0.279	0.006	0.191	0.062
Lung tissue	−0.393	<0.001	−0.653	<0.001	0.381	<0.001	0.531	<0.001

Abbreviations: *LINE*-*1*, long interspersed nucleotide element; *iNOS*, inducible nitric oxide synthase; *APC*, adenomatous polyposis coli; H3K9ac, H3K9 acetylation.
