# Supplementary material for: Dose- and time- effect responses of DNA methylation and histone H3K9 acetylation changes induced by traffic-related air pollution
Source: Sci Rep. 2017 Mar 3;7:43737. doi: 10.1038/srep43737 (PMC5335614; doi:10.1038/srep43737)
Supplement: Supplementary Information [file srep43737-s1.pdf]

Dose- and time- effect responses of DNA methylation and histone H3K9 acetylation changes induced by traffic-related air pollution

Authors: Rui Ding[1,4], Yongtang Jin[1*], Xinneng Liu[1], Huaizhuang Ye[1], Ziyi Zhu[2], Yuan Zhang[1], Ting Wang[1], Yinchun Xu[3]

Supplementary figures

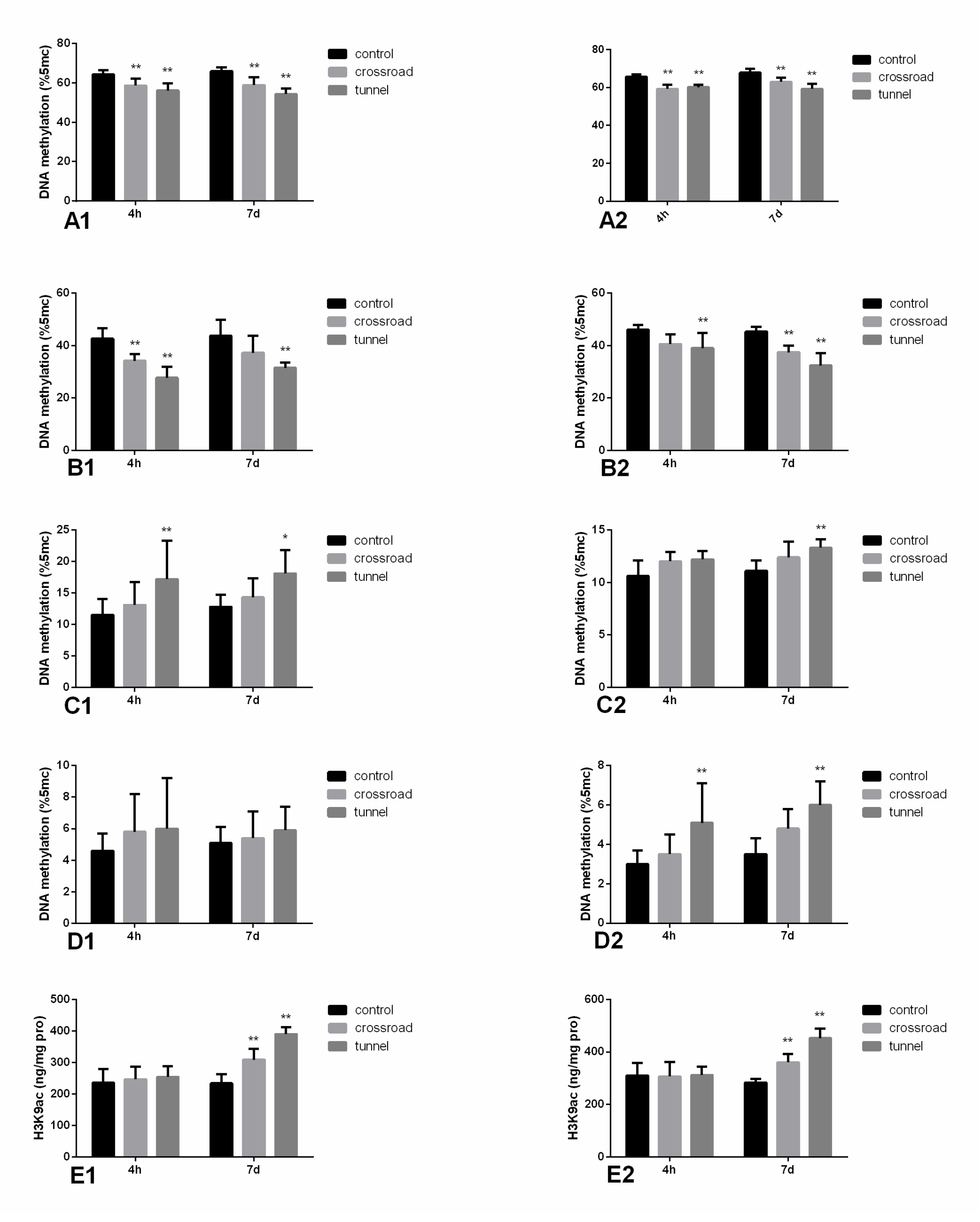

Supplementary Figure S1 Comparison of DNA methylation and H3K9ac after exposed for 4 h and 7 d at different exposure sites.

A1, *LINE-1* methylation in blood DNA; A2, *LINE-1* methylation in lung tissue DNA; B1, *iNOS* promoter methylation in blood DNA; B2, *iNOS* promoter methylation in lung tissue DNA; C1, $p16^{CDKN2A}$ promoter methylation in blood DNA; C2, $p16^{CDKN2A}$ promoter methylation in lung

tissue DNA; D1, *APC* promoter methylation in blood DNA; D2, *APC* promoter methylation in lung tissue DNA; E1, H3K9ac in PBMC histone; and E2, H3K9ac in lung tissue histone.

$^{*}P<0.05$, $^{**}P<0.01$, comparing with the corresponding control group.

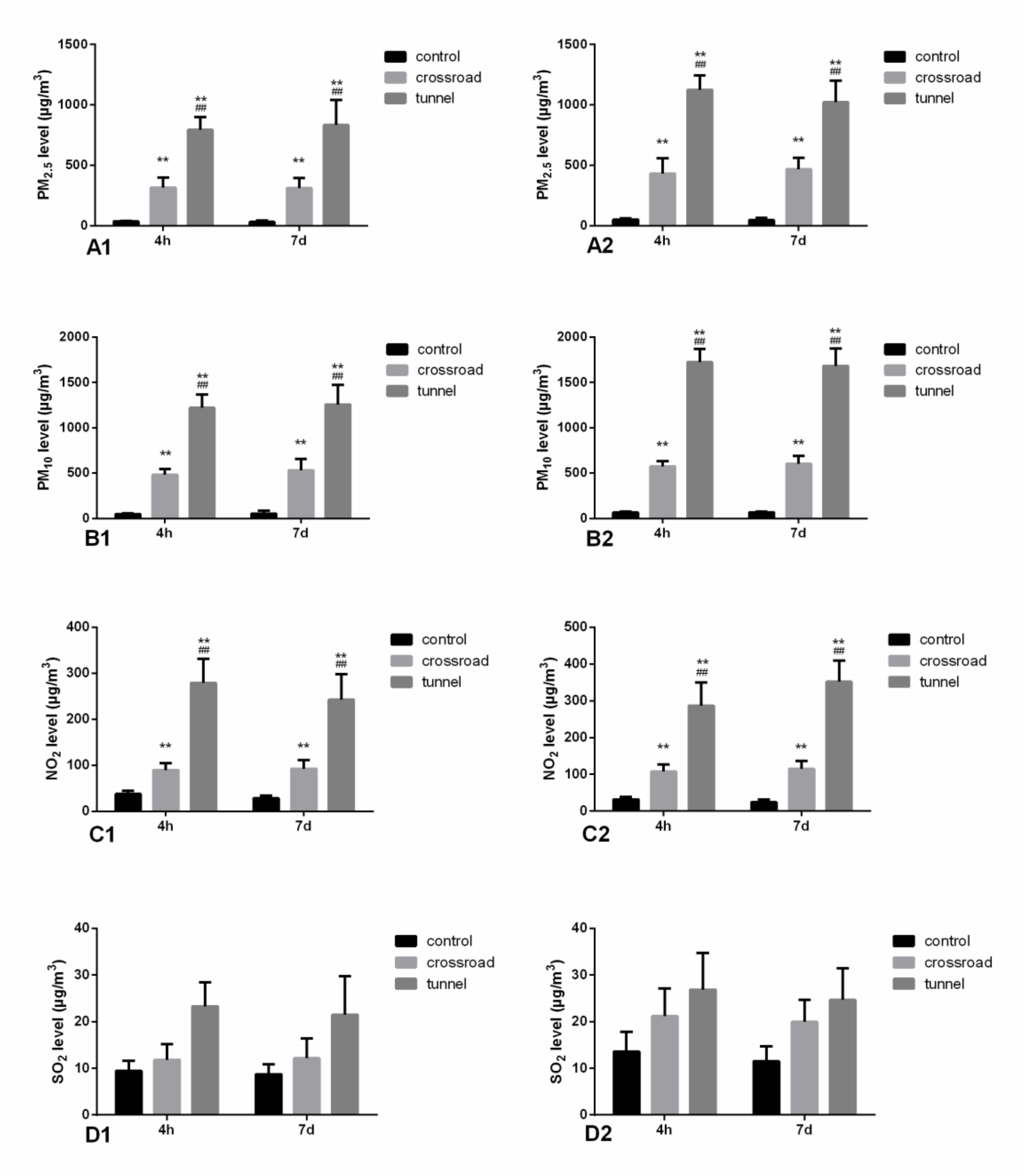

Supplementary Figure S2 Comparison of air pollutants at the different exposure sites per season.

A1, $PM_{2.5}$ level in spring; A2, $PM_{2.5}$ level in autumn; B1, PM_{10} level in spring; B2, PM_{10} level in autumn; C1, NO_2 level in spring; C2, NO_2 level in autumn; D1, SO_2 level in spring; and D2, SO_2 level in autumn.

$^{**}P<0.01$, comparing with the corresponding control group.

$^{\#\#}P<0.01$, comparing with the corresponding crossroad group.
